# Commentary: Dignity of older persons with mental health conditions: why should clinicians care?

**DOI:** 10.3389/fpsyt.2025.1553697

**Published:** 2025-04-03

**Authors:** Hideki Muramatsu

**Affiliations:** Graduate School of Arts and Sciences, The Open University of Japan, Chiba, Japan

**Keywords:** dignity, human rights, the elderly, ageism, mental health, recovery

## 1 Introduction

Banerjee et al. (1) highlighted the importance of dignity as a central component of mental health care for the elderly. They also emphasized the need for research addressing mental health interventions [including dignity therapy (DT)] in the context of ageism, rights, respect, and equality. This paper builds on the work of Banerjee et al. (1) by introducing the culturally sensitive framework of flat recovery process (FRP) (2) into recovery-oriented and person-centered practices to further the dialogue regarding the necessity for service providers to consider and incorporate human dignity into the care of their clients. This paper is also an educational resource for mental health care professionals working with geriatric populations. This commentary serves as an innovative material for fostering constructive discussions on human dignity through the lens of cultural diversity.

## 2 Conceptual use of ableism

An increasing proportion of the elderly experience mental illness, including chronic medical conditions, geriatric depression (3, 4), anxiety (4), and poorer cognitive functioning (4, 5). Concepts such as independence, respect, autonomy, and rights are crucial for functional recovery and optimal management, most of which originate in Western paradigms (1). The need for a global paradigm of mental health care for the elderly that supports their universal human dignity is growing (1), in support of their dignity as universal dignity. Interventions such as DT, aligned with the United Nations Sustainable Development Goals for 2030 (6), may address this need.

The definitions of ageism are broad and cover interdependent and distinct qualities from ableism (7). Disability is cast as a diminished state of being human (8), whereas ableism is discrimination against diminished human abilities. Furthermore, as aging is associated with a change in mental and physical abilities, the connotations of being elderly tend to be negative (9). Accordingly, this study focuses on the non-able nature of ageism and ableism.

To protect universal human dignity while respecting cultural diversity, the current article introduces the concept of FRP for mental disorders (2), a framework to serve as an educational resource (1) for mental health care professionals working with the elderly, to hopefully facilitate constructive discussions. FRP introduces a different perspective on the recovery process, shifting attention away from upward and downward mobility to a less hierarchical perspective. As time passes, one naturally moves deeper into the interior of their psyche, resulting in change or development across their lifespan (2). The downward shifts in life do not represent backsliding but rather a normal change or development across the lifespan.

The FRP reinterprets, aligned with the United Nations Universal Declaration of Human Rights (UDHR) (10), and rejects the evaluation of human worth based on superiority or inferiority during life’s *ups and downs* (11) or diminished abilities across the lifespan. It challenges the notion that aging represents a *descent* into a less valuable state within life’s *ups and downs* (11). The change in physical and cognitive functions due to aging can cause various *non-able* biases. Ageism exacerbates the stigma of mental illness in neurodegenerative diseases and is also associated with the stigma of dementia and mental illness (1). These biases often stratify individuals into hierarchies of superior and inferior abilities based on diminished capacities due to aging or mental illness, calling for specialized education and training for medical and social professionals to address individual psychological barriers and social obstacles stemming from ableism. The concept of FRP is meaningful for elderly people with mental illnesses and as a human rights perspective on human life and existence that does not define the meaning of existence based on arbitrary human authority. Therefore, the concept of the FRP should be introduced into discussions on ageism.

The FRP reinterprets recovery from a social constructivist perspective (12) to mitigate feelings of guilt or shame from a perspective that does not evaluate humans, whatever their illnesses or age, in terms of superiority or inferiority. It seeks to reduce ableism by emphasizing a non-hierarchical view of human abilities. Aging is a challenging process (13) involving physical and cognitive change (1), often undervalued or overlooked by society (13). Introducing the concept of the FRP into ageism may alleviate the ableist climate in which human values change in the life process in relation to the change of physical, mental, and cognitive abilities. Tackling these issues may help combat ageism.

For instance, the recovery process for individuals with mental disabilities acknowledges that everyone experiences crises (14). From the perspective of the life process, the change in capabilities of the elderly has commonality with the discussion on “non-able” in terms of impairment or mental illness. As Muramatsu (15) states: “Humans are existential beings, finite, and in constant flux as they interact with the environment. This leads to a change in physical functioning, aging, cognitive change, dementia, and ultimately death” (p. 14). Their shared dignity does not evaluate human worth based on ability or productivity (2). The FRP concept (2), developed from the perspective of an expert-by-experience (2, 15, 16), can be used as an educational resource to expand a reader’s responsiveness profile, a comprehensive list of factors to which an individual responds (17).

## 3 Discussion

Considering universal human dignity in the context of ageism and ableism, as outlined in the UDHR (10), requires recognizing that egalitarian strategies must coexist with hierarchical strategies (18). This article supports the approach of Banerjee et al. (1), which emphasizes human dignity. Furthermore, introducing ableism and ageism supports this dignity-centered perspective and may contribute to generating a new global outlook for mitigating and balancing hierarchical strategies. Although this paper does not delve deeply into every aspect, it serves as a meaningful introduction to concepts related to ableism. It emphasizes the importance of improving clinical empathy among mental health professionals (15) and medical staff caring for older adults (1, 19). Additionally, academia needs to teach and train people (20) to address the overlapping dangers of ageism and ableism (21), fostering mutual respect in interpersonal relationships. I believe this article serves as a meaningful introduction to concepts related to ableism and makes a modest contribution toward advancing the discussion of human dignity (1) within the social structure, particularly as an educational resource. I hope that new findings from various interdisciplinary studies will further promote the ideal of universal human dignity, transcending the confines of age-related frameworks.
